# Toward Accurate Cybersickness Prediction in Virtual Reality: A Multimodal Physiological Modeling Approach

**DOI:** 10.3390/s25185828

**Published:** 2025-09-18

**Authors:** Yang Long, Tieyan Wang, Xiaoliang Liu, Yujiang Li, Da Tao

**Affiliations:** 1 Institute of Human Factors and Ergonomics, College of Mechatronics and Control Engineering, Shenzhen University, Shenzhen 518060, China; yang_long_szu@163.com (Y.L.);; 2 SDIC Intelligent Xiamen Information Co., Ltd., 188 Qianpu East Road, Xiamen 361008, China

**Keywords:** cybersickness, physiological measures, machine learning, multimodal modeling, virtual reality

## Abstract

**Highlights:**

**What are the main findings?**

- EDA-based regression models outperformed ECG-based and multimodal models in VR cybersickness prediction, with Ensemble Learning achieving a maximum R^2^ of 0.98.
- SC mean, SC max, SC variance, SDNN, and HRMAD were identified as key features in physiological-signal-based VR cybersickness prediction.

**What is the implication of the main findings?**

- This study provides an important reference for developing accurate and interpretable cybersickness prediction models and assessment systems in VR.
- The findings offer valuable guidance for optimal selection of physiological features and sensors in cybersickness assessment systems.

**Abstract:**

Cybersickness poses a significant challenge to the widespread adoption of virtual reality (VR), as it impairs user experience and operational performance. This study proposes a physiological modeling approach to objectively assess cybersickness severity during VR experience. An interactive VR experiment was conducted, inducing varying levels of cybersickness through VR navigation tasks under different field-of-view and graphic quality settings. Physiological signals (i.e., electrodermal activity (EDA) and electrocardiogram (ECG)) were continuously recorded and extracted to build multiple machine learning regression models for cybersickness prediction. The results showed that EDA-based models consistently outperformed ECG-based models across all algorithms, with the Ensemble Learning model achieving the highest predictive accuracy (R^2^ = 0.98). In contrast, ECG-based models yielded limited predictive capability (R^2^ = 0.53). Combining ECG with EDA features showed little improvement in model accuracy, suggesting a limited complementary role of ECG features. SHAP-based feature importance analysis revealed that EDA features (e.g., mean, maximum, and variance of skin conductance) were the most effective features in cybersickness prediction, which captured both tonic arousal and phasic autonomic responses during the cybersickness process. ECG features such as SDNN and HRMAD contributed modestly, offering physiological interpretability despite being less effective in cybersickness prediction. The findings demonstrate the feasibility of using low-burden physiological signals for accurate and interpretable prediction of cybersickness severity. The proposed approach supports the development of lightweight, real-time monitoring systems for VR applications, offering practical advantages in terms of simplicity, adaptability, and deployment potential.

## 1. Introduction

Virtual reality (VR) has emerged as a key medium for immersive interaction, with wide-ranging applications in education and training, medical rehabilitation, military simulation, and entertainment gaming [1,2,3]. Through realistic visual rendering and high interactivity, VR environments offer users a strong sense of presence and immersion. According to The Business Research Company [4], the global VR market is projected to grow at a compound annual growth rate of 12.9%, reaching USD 28.34 billion by 2029. However, this enhanced immersive experience also introduces significant challenges in physiological adaptation. Many users experience discomfort symptoms such as nausea, dizziness, and eye fatigue when using VR devices. These symptoms can be collectively referred to as cybersickness [5], which poses a significant barrier to the widespread adoption of VR technology.

Cybersickness is commonly regarded as a subtype of motion sickness, primarily caused by sensory conflicts among the visual, vestibular, and somatosensory systems. Unlike traditional motion sickness, typically driven by vestibular input, cybersickness is largely triggered by visually induced conflicts, especially when using head-mounted displays [6]. These symptoms not only diminish users’ sense of immersion and willingness to engage but also impair task performance and may even cause users to terminate VR use prematurely [7]. Research has shown that more severe cybersickness is associated with slower reaction time, reduced accuracy, and elevated physiological stress [8,9]. Consequently, accurate and real-time assessment of cybersickness is critical for developing effective interventions aimed at alleviating or eliminating its symptoms, thereby ensuring a high-quality user experience and enhancing system adaptability.

Traditionally, cybersickness can be assessed by three methods: subjective questionnaires, task performance metrics, and physiological measurements. Specifically, the Simulator Sickness Questionnaire (SSQ) [10] and the Virtual Reality Sickness Questionnaire (VRSQ) [11] are commonly used questionnaires to capture users’ subjective discomfort. However, subjective questionnaires are typically administered after task exposures or at specific time points, thereby lacking real-time capability and limiting support for dynamic system interventions. Task performance metrics, such as reaction time and operational accuracy, can partially reflect functional impairment but are susceptible to individual differences and task complexity, resulting in limited stability and sensitivity [12]. In contrast, physiological signals offer a promising avenue for assessing cybersickness by capturing users’ autonomic and affective responses to virtual environments. Modalities such as electroencephalography (EEG), electrodermal activity (EDA), and electrocardiogram (ECG) have been explored in prior research. EEG reflects cortical responses to sensory conflict, but its practicality is limited by equipment complexity and sensitivity to motion artifacts [13]. EDA provides a sensitive index of sympathetic arousal [14], while ECG enables the extraction of heart rate and heart rate variability (HRV), reflecting autonomic regulation [15]. Compared to EEG, both EDA and ECG are more robust to motion and easier to implement in dynamic VR settings. Their physiological relevance and technical feasibility make them promising candidates for low-burden and accurate cybersickness assessment.

Although early studies have demonstrated associations between many physiological measures and cybersickness [13,14,15], there is no single true measure that could be universally valid in assessing cybersickness across varied contexts. Each physiological measure may reflect only limited and distinct aspects of the physiological responses associated with cybersickness, and they thus cannot fully capture its multidimensional nature. To address this limitation, recent research has applied machine learning techniques to integrate multiple signals and model their complex interactions. For example, Qu, et al. [16] induced cybersickness through a VR passive navigation task and collected EDA, ECG, and avatar posture data. Using an LSTM–Attention model, they achieved an accuracy of 96.85% in cybersickness classification. Shimada, et al. [17] applied deep learning to short-term eye-tracking data collected during VR scenarios involving car and roller coaster simulations, successfully classifying four levels of cybersickness severity with up to 80% accuracy in personalized models. Sameri, et al. [18] combined EDA, EEG, photoplethysmography (PPG), and skin temperature signals within a supervised learning framework, achieving 86.66% accuracy in predicting elevated cybersickness symptoms during a VR roller coaster simulation. Collectively, these findings support the feasibility of using multimodal physiological signals for effective cybersickness assessment.

Although previous research has made notable progress, several critical limitations remain. First, while various physiological signals have been explored for cybersickness assessment [16,18], systematic evaluation of low-burden, head-mounted-display-compatible modalities (e.g., EDA and ECG) is scarce. For example, Chang, et al. [19] reviewed EEG-based cybersickness assessment studies and noted that most studies used passive scenarios (e.g., driving, navigation), with reported accuracies between 79% and 100%. However, EEG-based approaches are often less practical due to cumbersome setup, susceptibility to motion artifacts, and reduced user comfort. In contrast, the predictive value of EDA and ECG in interactive VR contexts has received limited investigation. Second, many prior models lack interpretability [16,17], making it difficult to identify which specific physiological features contribute most to prediction or to understand the underlying mechanisms linking these signals to cybersickness. Third, the majority of existing studies employ categorical classification of symptom severity [16,19], which constrains the precision of cybersickness assessment and fails to reflect the continuous variation in cybersickness symptoms during immersive VR experience. Regression-based approaches, however, have been rarely explored in this context, and their adoption could provide more precise, continuous estimation of cybersickness severity, offering more ecologically valid insights into users’ moment-to-moment experiences in VR. Addressing these gaps requires predictive frameworks that combine practical physiological modalities with interpretable modeling techniques capable of capturing continuous changes in cybersickness severity.

To address these research gaps, the present study proposes a regression-based machine learning approach for predicting cybersickness severity using physiological signals in VR environments. Given the limited compatibility of EEG systems with head-mounted displays, we selected EDA and ECG as the input modalities due to their robustness and practical applicability. While previous studies mainly adopted passive viewing VR scenarios [19], which might fail to capture the interactive dynamics of users’ VR experience and thus have limited ecological validity, we designed an interactive VR experiment with a 4 × 4 factorial combination of field-of-view (FOV) and graphic quality levels, where participants completed active navigation tasks under varying VR conditions. EDA and ECG signals were continuously recorded during task performance. We developed regression models using six representative machine learning algorithms and compared their performance across unimodal (EDA or ECG) and multimodal (EDA + ECG) configurations. To improve model interpretability and clarify the specific contributions of physiological signals, SHAP (SHapley Additive exPlanations)-based feature importance analysis was also performed. The proposed approach provides empirical evidence that could support the feasibility of accurate, interpretable, and low-burden cybersickness prediction, offering practical value for real-world VR applications.

## 2. Methodology

### 2.1. VR Cybersickness Experiment

#### 2.1.1. Participants

Thirty participants (fourteen females; mean age = 22.9 years, SD = 1.6) participated in the experiment. All participants were right-handed, had normal or corrected-to-normal vision, and none reported musculoskeletal or neurological disorders. Thirteen participants (42%) reported no prior experience with VR, while the rest had limited exposure to VR. Regarding cybersickness history, 45% had never experienced screen-induced dizziness, while the rest reported dizziness induced by mobile phones (24%), computers (30%), VR headsets (30%), or television (30%). The study was approved by the Ethics Committee of Shenzhen University, and informed consent was obtained from all participants prior to their involvement.

#### 2.1.2. Experimental Design and Task

Previous studies have indicated that FOV and virtual graphic quality are two important visual display parameters that are known contributors to the onset and intensity of cybersickness [20,21]. In this study, FOV, defined as the horizontal viewing angle in the VR environment [20], was set at 90°, 120°, 150°, and 180°, following prior research suggesting that FOVs below 90° reduce immersion, while those above 180° exceed the natural human field of view [22]. A 30° increment was chosen to balance experimental feasibility with the ability to capture continuous effects of FOV on cybersickness. Graphic quality was operationalized through video resolution, which directly affects image clarity, texture fidelity, and visual realism. Four levels of resolution were selected based on standards outlined by the Society of Motion Picture and Television Engineers (SMPTE): 480p (916 × 480), 720p (1375 × 720), 1080p (2068 × 1080), and 4K (3664 × 1920).

Participants completed VR tasks under 16 experimental conditions covering all combinations of graphic quality and FOV. The task, presented in first-person view, comprised two subtasks: ball collection and tracing. In the ball collection task, participants needed to locate and pick up red balls randomly distributed throughout a virtual maze. The tracing task required participants to quickly move to a newly appeared target at a random location after each successful collection. Each task lasted 100 s, during which participants were instructed to collect as many balls as possible. These tasks incorporated typical VR activities such as target search, path planning, and spatial navigation, and these have been shown to effectively induce cybersickness [23,24].

#### 2.1.3. Apparatus and Data Collection

The experimental VR environment was developed in Unity3D (Unity Personal 2022.3.15) and deployed via SteamVR [25]. The VR environment was presented with the mainstream VR device PICO NEO3 (Qingdao Xiaoniao Kankan Technology Co., Ltd., Qingdao, China; screen resolution: 3664 × 1920 pixels; refresh rate: 90 Hz; 6 GB RAM; 256 GB storage), combined with its dedicated controllers to support intuitive interactions within the virtual environment. ECG and EDA signals were synchronously recorded using the ErgoLab 3.0 system (Kingfar, Beijing, China), which integrated the corresponding physiological sensors. Specifically, ECG was acquired via a standard three-lead configuration at 1024 Hz, with electrodes placed on the left clavicle, left mid-axillary line, and the 5th intercostal space [26]. EDA was sampled at 64 Hz, with electrodes attached to the volar pads of the left-hand index and middle fingers [27]. Participants’ subjective perception of cybersickness was assessed using the VRSQ, which provides a standardized total score ranging from 0 to 100, with higher scores indicating more severe symptoms [11]. This total score was used as the ground truth label for subsequent model training.

#### 2.1.4. Procedures

**Figure 1:**
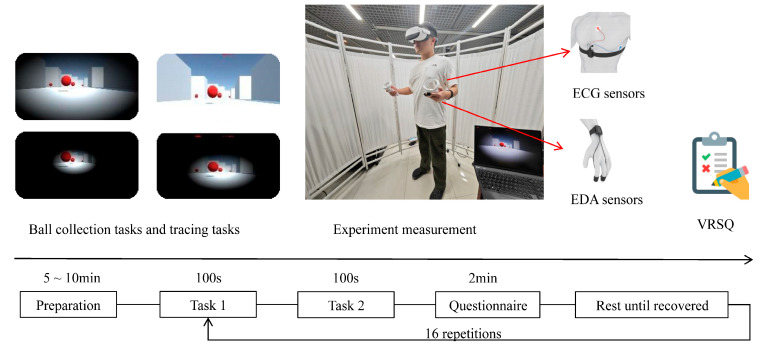
Procedures of the cybersickness experiment.

Participants first completed a demographic questionnaire and signed an informed consent form. They were then briefed on the experiment and were guided to wear the physiological sensors according to standardized operating procedures [16]. A pilot test was conducted to confirm that the lightweight, wearable EDA and ECG sensors used in our study would not interfere with participants’ task performance or natural responses. After a short acclimation session to get familiar with the VR system, tasks, and physiological sensors, participants were allowed sufficient time to adapt to the devices before the formal experiment began. Participants then performed the 16 experimental conditions in a Latin square order to counterbalance possible order effects. After each experimental condition, they were required to complete the VRSQ and rested for at least five minutes to minimize potential discomfort and fatigue. Physiological signals were recorded synchronously throughout the experiment (Figure 1).

### 2.2. Machine Learning Modeling

#### 2.2.1. Data Preprocessing and Feature Extraction

This study adopted widely used physiological signal preprocessing techniques reported in the prior literature [28,29,30]. For ECG signals, wavelet denoising was first applied, followed by a bandpass filter (1–200 Hz) and a Butterworth notch filter (49–51 Hz) to remove baseline drift and powerline interference [29]. To correct abnormal heartbeat intervals, identified irregular R-R intervals were handled using mean interpolation. For EDA signals, a bandpass filter (0.02–0.2 Hz), wavelet denoising, and Gaussian smoothing were employed to suppress baseline drift and sudden noise artifacts [30]. Subsequently, the preprocessed signals were segmented using a sliding-window approach. For each 100 s task, the first 10 s was discarded to eliminate potential transient effects at the beginning of the trial, leaving 90 s of valid data for feature extraction. Then, the 90 s signals were segmented into 32 samples, which were linked to the VRSQ score reported after the task performance under the experimental condition. After aggregating across all conditions and participants, and excluding outliers, a total of 30,000 samples were obtained for subsequent machine learning analyses. For ECG, common HRV metrics were extracted, including SDNN, RMSSD, pNN20, and pNN50, reflecting the dynamics of the autonomic nervous system (Table 1) [31]. For EDA, features such as skin conductance (SC) and skin conductance level (SCL) were included, which are widely used to characterize physiological responses under motion sickness or mental stress [14,16,32]

To reduce feature redundancy and improve modeling efficiency, the Minimum Redundancy Maximum Relevance (MRMR) algorithm was employed to rank and select features separately for ECG, EDA, and the fused modality (ECG + EDA) modeling. This method considers both feature relevance to the target variable and inter-feature redundancy, facilitating the selection of a representative feature subset [33]. Ultimately, the following representative features were retained and used in subsequent modeling for different modalities: 10 features for ECG-based modeling, 6 features for EDA-base modeling, and 18 features for the fused modality modeling (Table 1). Before modeling, all features were normalized to eliminate the influence of differing scales on model training.

#### 2.2.2. Regression Modeling and Evaluation

This study formulated cybersickness prediction as a continuous regression task, with VRSQ scores as the target variable. To this end, six representative regression models were constructed: Linear Regression, Decision Tree regression, Kernel-based Regression, Ensemble Learning, Neural Networks, and Gaussian Process Regression. These methods cover a broad spectrum of modeling paradigms, from linear to nonlinear, and from parametric to non-parametric, allowing for a systematic evaluation of the predictive potential of multimodal physiological signals.

Linear Regression, incorporating interaction terms to account for potential feature interactions, was selected for its interpretability and suitability for approximately linear data distributions [34]. Decision Tree regression was adopted to model nonlinear relationships by recursively partitioning the feature space [35]. Ensemble Learning included two representative methods: bagging-based random forests and boosting-based gradient trees; the former reduces variance through bootstrap aggregation, while the latter improves prediction by sequentially refining weak learners [36]. Neural Networks with one or multiple hidden layers were used to capture complex nonlinear mappings inherent in physiological data [37]. Gaussian Process Regression was selected for its capacity to provide both predictive estimates and uncertainty quantification within a Bayesian nonparametric framework, particularly advantageous under small-sample and noisy conditions [38]. Kernel-based Regression included support vector regression and least squares kernel regression, both of which model complex nonlinear relationships by projecting data into high-dimensional feature spaces [39].

All models were trained using features extracted from the three input modalities: ECG, EDA, and fused ECG + EDA. Hyperparameters were tuned using a grid search strategy [40]. Specifically, for Ensemble Learning, boosting trees were configured with a maximum split of 20 and bagging trees with a maximum split of 200. Decision Tree regression included fine, medium, and coarse trees with maximum splits of 100, 20, and 4, respectively. Kernel-based Regression included SVM and logistic regression learners, while Gaussian Process Regression employed various kernel functions (squared exponential, Matern 5/2, exponential, and rational quadratic). Linear Regression variants included standard linear, interaction, robust, and stepwise models (maximum steps = 1000). Neural Networks were tested with different architectures, including narrow (10 neurons), medium (25 neurons), wide (100 neurons), two-layer (10-10 neurons), and three-layer (10-10-10 neurons) fully connected networks. Model performance was evaluated using root mean square error (RMSE), mean squared error (MSE), mean absolute error (MAE), and coefficient of determination (R^2^), averaged across ten-fold cross-validation. For algorithm families with multiple variants, the model achieving the best average performance was selected for reporting and further analysis.

To enhance model interpretability, SHAP analysis was conducted to identify the most influential physiological features contributing to cybersickness prediction. The Bagging Regressor, an Ensemble Learning method based on bootstrap aggregation, was selected for this analysis as it consistently achieved either the best or second-best performance across all three input modalities and is well-suited for SHAP computation due to its robustness to feature scaling.

## 3. Results

### 3.1. Modeling Results

**Figure 2:**
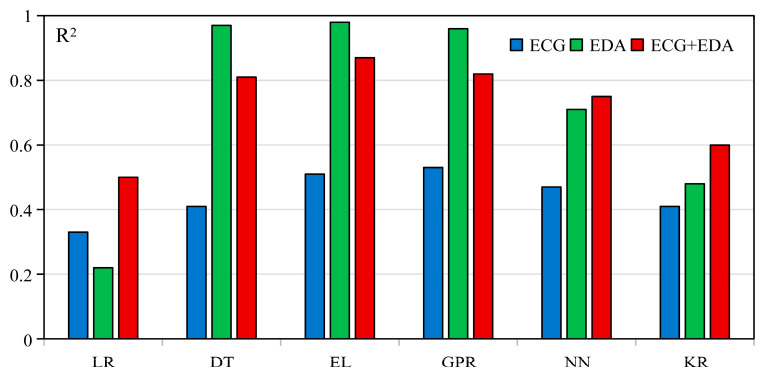
R^2^ of different modalities across models.

Table 2 presents the modeling outcomes for three physiological input modalities: ECG, EDA, and the fusion of ECG and EDA. Overall, the models demonstrated varying degrees of predictive performance depending on the physiological modality and modeling approach. EDA-based models achieved the most accurate predictions across evaluation metrics, especially when using Ensemble Learning approaches (R^2^ = 0.98, MAE = 0.04, MSE = 0.02, RMSE = 0.15). This suggests that EDA contains rich information relevant to cybersickness responses. In contrast, ECG-based models demonstrated limited predictive capability, with relatively low R^2^ values across algorithms. This indicates that ECG alone may not sufficiently capture the physiological dynamics associated with cybersickness. The fusion of ECG and EDA yielded more stable performance compared to ECG alone and showed advantages in some model configurations. However, fusion did not consistently outperform EDA across all modeling conditions. This indicates that the added value of signal fusion is contingent on the complementarity of signal modalities and their compatibility with the model architecture. These findings indicate that the predictive effectiveness of cybersickness models is influenced more by the informativeness of the physiological modality than by the choice of algorithm. Figure 2 illustrates the distribution of R^2^ scores for all combinations, providing an overview of model performance across input conditions.

### 3.2. Feature Importance Across Different Modalities

**Figure 3:**
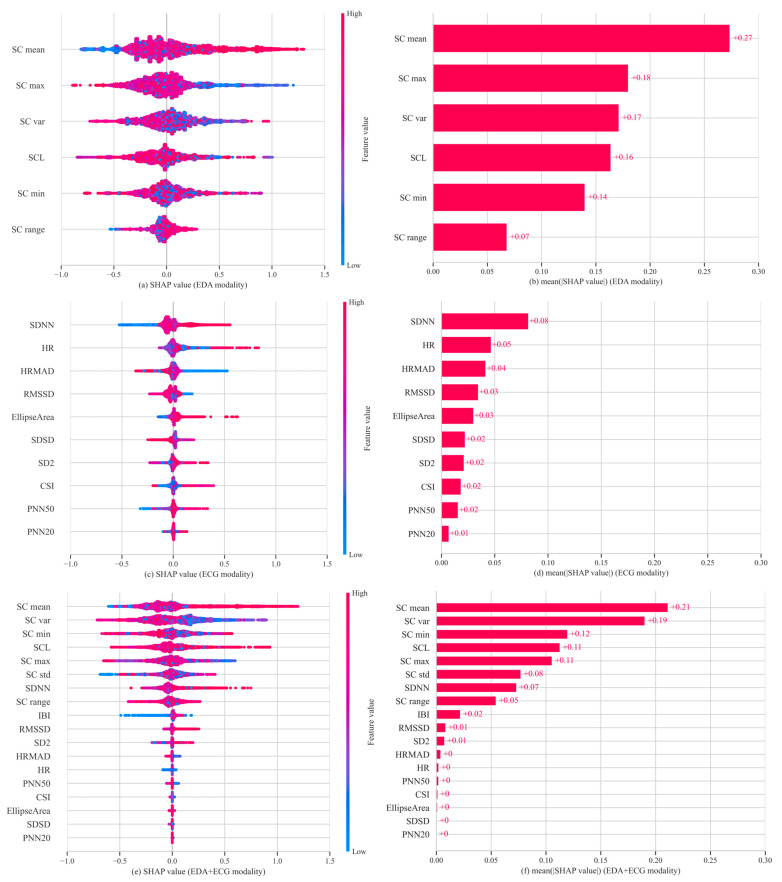
Feature importance analysis across three physiological modalities. (**a**) SHAP summary plot for EDA; (**b**) mean absolute SHAP values for EDA; (**c**) SHAP summary plot for ECG; (**d**) mean absolute SHAP values for ECG; (**e**) SHAP summary plot for EDA + ECG; (**f**) mean absolute SHAP values for EDA + ECG.

**Figure 4:**
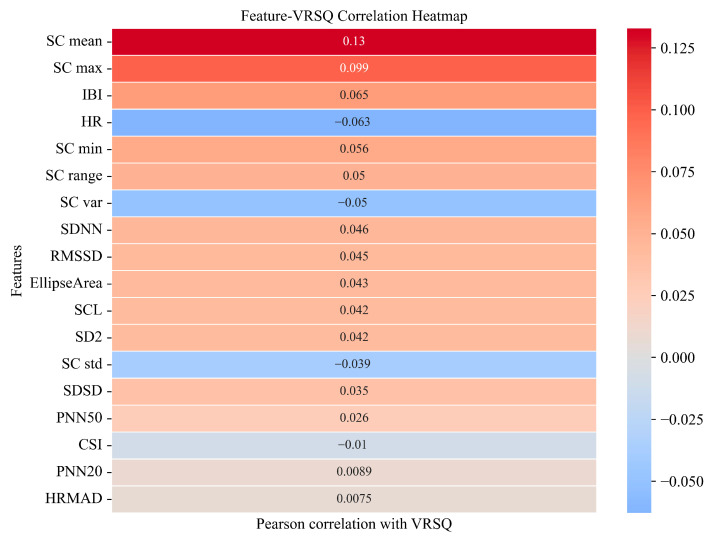
Pearson’s correlation heatmap between physiological features and VRSQ scores. Features are ordered from top (strongest) to bottom (weakest). Red indicates positive correlations, and blue indicates negative correlations.

Figure 3 presents the SHAP summary plots of feature importance for the best-performing unimodality and dual-modality models. These visualizations illustrate how individual features affected model outputs and highlight their relative contributions to cybersickness prediction. In the ECG modality, SDNN emerged as the most important feature, followed by HR and HRMAD. However, overall SHAP values in this modality were relatively low, with the highest contribution value being approximately 0.08, indicating limited sensitivity of ECG features in capturing changes in cybersickness. In contrast, for the EDA modality, SC mean exhibited the highest SHAP value. Other features, except for SC range, also showed moderate and relatively balanced contributions. In the dual-modality setting, SC mean remained the most dominant contributor to model output. EDA features consistently showed high importance, while ECG features contributed less prominently. Among ECG features, only SDNN showed a SHAP value comparable to that of SC range. These results align with the modeling outcomes, reinforcing that EDA signals are more informative and sensitive for predicting cybersickness, especially in capturing subtle variations in user responses. In addition, Figure 4 presents the Pearson correlation heatmap between physiological features and VRSQ scores. The features are ranked by the absolute value of their correlation coefficients. Consistent with the SHAP results, EDA features such as SC mean (r = 0.13) and SC max (r = 0.099) exhibited the strongest associations with cybersickness severity. In contrast, ECG features showed generally weaker associations with cybersickness severity (e.g., HR (r = −0.063) and SDNN (r = 0.046)).

## 4. Discussion

This study designed a cybersickness-inducing experiment in a VR environment and collected participants’ multimodal physiological signals, including EDA and ECG. Based on the acquired data, multiple machine learning regression models were constructed to evaluate the predictive value of different physiological modalities in estimating cybersickness. In particular, both unimodal (EDA or ECG) and bimodal (EDA + ECG) input models were systematically compared across six mainstream regression algorithms. In addition to modeling performance, the relative importance of physiological features was also examined to better understand the underlying physiological mechanisms and modeling methods for cybersickness. The following sections provide a detailed discussion of model performance, underlying mechanisms, and practical implications in the context of the existing literature.

### 4.1. Evaluation of Unimodal and Bimodal Regression Models

Among the three input modalities, EDA-based models demonstrated the most robust predictive capability. Specifically, the EDA-based Ensemble Learning model achieved an R^2^ of 0.98, outperforming all other configurations. Except for Linear Regression, all EDA-based models consistently outperformed ECG models across multiple evaluation metrics, including RMSE, MAE, and MSE. Notably, Ensemble Learning, Decision Tree, and Gaussian Process Regression achieved R^2^ values above 0.9 based on EDA features. These findings provide compelling evidence that EDA, a well-established marker of sympathetic arousal, holds substantial predictive potential for real-time prediction of cybersickness severity. While prior studies have identified correlations between EDA and cybersickness onset or severity [14], most have used EDA in a supplementary role within EEG-based classification tasks [41]. As a result, EDA’s standalone predictive power in cybersickness assessment has been largely underexplored [16,18]. In fact, EDA is less invasive and easier to acquire compared to EEG and ECG, making it suitable for standalone continuous prediction. Thus, our study fills the research gap by systematically evaluating EDA across multiple machine learning algorithms and confirming its strength as a low-burden, non-invasive indicator for continuous cybersickness prediction.

In contrast, the predictive performance of ECG-based models was limited, with a maximum R^2^ of 0.53, significantly lower than that of EDA-based models. Although ECG is commonly used to track emotional and physiological states, its ability to capture variations in cybersickness severity appears limited. It may assist in detecting the onset of symptoms, but it lacks sufficient sensitivity to capture variations in severity [15,42]. This observation is consistent with previous studies that found only weak or inconsistent correlations between ECG-derived features and cybersickness severity [43,44]. These findings align with the present regression results and further confirm ECG’s limited standalone value for modeling continuous cybersickness responses.

While EDA showed clear advantages, integrating ECG and EDA signals produced mixed outcomes across different algorithms. For example, the bimodal model with Ensemble Learning reached a maximum R^2^ of 0.87, which is lower than that of its unimodal EDA counterpart (R^2^ = 0.98) but substantially higher than the unimodal ECG model (R^2^ = 0.51). Other algorithms, including Neural Networks, Kernel-based Regression, and Linear Regression, showed slight improvements with bimodal inputs; however, their maximum R^2^ reached only 0.75. These results indicate that while bimodal integration can enhance performance relative to weaker unimodal models, it does not necessarily surpass the strongest unimodal predictor when one modality is already highly informative. Previous studies have reported similar observations; for instance, Yang et al. [45] found that fusing EEG and ECG did not enhance cybersickness classification accuracy, and Liu et al. [46] observed that multimodal fusion without sufficient additional predictive value may not lead to substantial performance gains.

From the perspective of model selection, Ensemble Learning models consistently demonstrated superior performance in both unimodal and bimodal modeling settings. This result aligns with previous research by Sameri et al. [18], who emphasized the strengths of tree-based methods such as XGBoost and Decision Tree in handling physiological data with nonlinear characteristics. Zaidi et al. [47] also found tree-based models effective for capturing nonlinear and complex patterns, achieving over 93% accuracy in cybersickness classification. These models do not require assumptions about data distribution and effectively detect transient EDA features like skin conductance response peaks [48]. The hierarchical structure of tree-based models suits such physiological signals well, which likely explains their superior performance in this study. Beyond ensemble methods, Gaussian Process Regression also demonstrated competitive performance in this study. Specifically, Gaussian Process Regression achieved the highest R^2^ within the ECG unimodal setting (R^2^ = 0.53) and exhibited strong results with EDA (R^2^ = 0.96) and bimodal modeling (R^2^ = 0.82). These results suggest that Gaussian Process Regression is particularly well-suited for capturing the temporal dynamics and uncertainty inherent in physiological signals, owing to its probabilistic, non-parametric nature [49]. While ensemble-tree-based methods offered superior overall performance, the robust outcomes of Gaussian Process Regression highlight its potential as a complementary approach for modeling cybersickness with physiological data.

In summary, while bimodal models occasionally improved upon weaker unimodal models such as ECG, they generally did not exceed the performance of EDA-only models. Specifically, while the Ensemble-Learning-based bimodal model performed slightly worse than its unimodal EDA model counterpart, it showed considerable improvement over the unimodal EDA model. Overall, these findings emphasize that the predictive performance of multimodal models depends on the relative informativeness of each modality and highlight the importance of evaluating model outcomes empirically across algorithms.

### 4.2. Feature Importance and Physiological Mechanism Analysis

To further interpret the contribution of physiological features in model predictions, SHAP value analysis was performed using a stable Bagging Regressor to quantify the relative importance of each physiological feature. In the EDA unimodal model, SC mean showed the highest importance (mean SHAP value = 0.27), substantially exceeding that of other features. It also showed a positive correlation with VRSQ scores, suggesting that higher SC mean values reflect more severe cybersickness. This aligns with existing knowledge, as SC mean reflects the overall conductance level regulated by the sympathetic nervous system [14]. It typically increases with heightened arousal and is widely recognized as an indicator of emotional stress, cognitive load, or physical discomfort [50]. In contrast, SC var and SC max had lower importance, with mean SHAP values of 0.17 and 0.18, respectively, both negatively associated with predicted severity. These features likely reflect autonomic nervous system responsiveness by capturing short-term fluctuations and peaks in conductance. Such dynamics may reflect rapid sympathetic reactions and efficient physiological recovery, potentially mitigating subjective discomfort [50,51]. Conversely, individuals with persistently elevated SC mean but low variability may experience sustained arousal or reduced regulation capacity [52], reflecting physiological profiles associated with prolonged cybersickness.

Compared to EDA, the contribution of ECG features to the model was considerably lower. The most important feature, SDNN, had a SHAP value of 0.08 and showed a weak positive correlation with VRSQ scores. As a key HRV metric, SDNN reflects the balance between sympathetic and parasympathetic activity and is associated with autonomic stability [53]. While most studies report a negative relationship between SDNN and stress [54], our findings suggest that autonomic responses to cybersickness, primarily driven by visual–vestibular sensory conflict [6], may involve compensatory mechanisms or transient regulatory shifts under novel or low-intensity stimuli [55]. HRMAD exhibited a negative contribution, with higher values associated with lower predicted VRSQ scores. As a short-term HRV indicator, HRMAD reflects transient fluctuations in heart rate that may not represent sustained sympathetic activation. This suggests that such short-term variability could be associated with reduced cybersickness discomfort. HR showed inconsistent contributions, likely due to strong individual variability, and it thus lacks stable predictive value [43,44].

In the bimodal model combining ECG and EDA features, SHAP analysis revealed a feature importance pattern largely consistent with the unimodal results. The dominance of EDA features was even more apparent. SC mean continued to exert the strongest influence, while ECG features further declined in relative importance. This result has also been confirmed by the Pearson correlation analysis, which showed that EDA features, such as SC mean and SC max, exhibited strong associations with cybersickness severity, while ECG features (e.g., SDNN, HR and HRMAD) showed weaker correlations. Overall, these findings reinforce the robustness of EDA, particularly SC mean, as a physiological marker of cybersickness-related arousal.

Interestingly, while the bimodal (ECG + EDA) model achieved lower performance than the unimodal EDA model (R^2^ = 0.87 vs. 0.98), it performed substantially better than the unimodal ECG model (R^2^ = 0.53). This pattern suggests that the bimodal model represents an intermediate outcome, consistent with the relative predictive strength of the two modalities—EDA provides highly informative features, whereas ECG contributes weaker signals with limited complementary value. This interpretation is further supported by the Pearson correlation and SHAP analyses, which consistently showed stronger associations and more stable contributions for EDA features compared to ECG. While ECG features contributed less to prediction accuracy, certain HRV indicators such as SDNN and HRMAD offer valuable insights into autonomic dynamics. Together, these findings support the viability of EDA-based modeling for cybersickness assessment. They further highlight its potential for developing lightweight, EDA-based monitoring systems in VR environments.

### 4.3. Implications

This study advances the prediction of VR cybersickness by demonstrating that non-invasive, low-burden physiological signals can support continuous and practical assessment of user discomfort. Such capability enables VR systems to dynamically tailor visual and interaction parameters, improving comfort and sustaining immersion. By highlighting the deployment advantages of wearable-friendly modalities and providing guidance for effective multimodal integration, this work lays the groundwork for scalable, user-centered cybersickness mitigation solutions with strong potential for real-world adoption across diverse VR applications.

First, the findings provide actionable guidance for designing efficient, scalable, and low-burden cybersickness monitoring systems. EDA-based models consistently demonstrated robust predictive performance across multiple algorithms, confirming their utility as indicators of sympathetic nervous system activation. Compared to EEG systems, EDA sensors are low-cost, energy-efficient, and easily integrated into wearable platforms, making them well-suited for real-time applications with constrained computational or power budgets [16,56]. Prioritizing EDA acquisition may reduce system complexity without sacrificing model performance, thus supporting the development of lightweight, scalable VR systems.

Second, the findings highlight that multimodal fusion does not inherently guarantee improved performance. When EDA alone provides sufficient predictive information, incorporating ECG may introduce redundancy or noise that degrades model accuracy. This underscores the importance of selecting modalities based on physiological complementarity rather than quantity. Indeed, in many multimodal learning contexts, the informativeness and compatibility of signals have a greater impact on performance than the choice of learning algorithm [57]. These insights highlight that targeted signal integration, rather than indiscriminate modality expansion, is essential for optimizing model generalizability and efficiency.

Third, the study also contributes theoretical insights into the physiological underpinnings of cybersickness. SHAP-based interpretability analysis further revealed the distinct roles of key physiological indicators, such as SC mean, SC var, and SDNN, illustrating how both tonic sympathetic arousal and phasic autonomic responsiveness shape cybersickness symptomatology. Such mechanistic understanding enhances model transparency and can inform the design of adaptive interventions for user comfort regulation.

Moreover, this work demonstrates the value of formulating cybersickness prediction as a regression problem rather than a categorical classification task. While classification approaches have been widely adopted in prior research, they constrain assessment to discrete labels and overlook the continuous, dynamic nature of cybersickness symptoms. By contrast, regression enables fine-grained estimation of symptom severity, providing richer and more ecologically valid insights into user experience. This perspective reframes cybersickness modeling as a continuous prediction problem, positioning regression as a complementary paradigm that extends beyond the limitations of classification-based methods.

Taken together, these findings provide a foundation for developing dynamically adaptive and personalized cybersickness prediction systems. By enabling continuous monitoring of users’ physiological states, VR platforms can proactively adjust interface parameters to deliver personalized interventions, thereby enhancing user comfort and overall immersion. Furthermore, the proposed predictive approach offers a scalable and interpretable blueprint for next-generation wearable and embedded HCI systems, promoting the broader adoption of intelligent, user-aware VR solutions.

### 4.4. Limitations and Future Directions

While this study demonstrates the feasibility and potential of leveraging multimodal physiological signals for continuous cybersickness prediction, several limitations warrant consideration. First, our participants were young adults recruited from a university campus, resulting in a demographically homogeneous sample. This is likely to limit the generalizability of the proposed models to broader populations with different ages, VR experiences, or health conditions. Future research should include larger and more diverse samples to examine the influence of inter-individual differences on cybersickness prediction [58].

Second, this study focused exclusively on two physiological modalities (i.e., EDA and ECG). While these signals demonstrated promising predictive performance, both EDA and ECG require specialized wearable devices for measurement, such as fingertip sensors for EDA and electrodes placed on the body for ECG, which may affect user comfort and overall experience. Future research seeking to improve user experience could incorporate wearable-compatible physiological signals such as eye tracking (ET) and respiration (RESP) [59,60]. By exploring more advanced multimodal fusion strategies, these additions hold promise for developing monitoring solutions that are not only more robust but also less invasive and more user-friendly than EDA and ECG modalities, thereby facilitating wider adoption in real-world applications.

Third, the experimental scenarios were limited to two tasks (ball tracking and collection). While these tasks were selected based on prior research and cover core VR interaction modes, they differ from the diverse real-world VR applications (e.g., VR driving simulations, VR surgical training). Consequently, the generalizability of our findings to other VR tasks or more complex environments may be limited. Future studies should consider incorporating a broader variety of VR scenarios to evaluate the robustness and applicability of cybersickness prediction models across different interaction contexts.

Finally, the modeling framework in this study was based on conventional machine learning techniques and employed subject-dependent cross-validation rather than a subject-independent validation strategy. As a result, the reported performance may in part reflect the learning of person-specific physiological patterns rather than generalizable responses to cybersickness, thereby limiting the models’ applicability across diverse users. Future research could explore optimization strategies, such as transfer learning and personalized modeling, to more effectively address inter-individual differences [61], thereby enhancing the personalization and adaptability of cybersickness prediction models, especially when applied to more diverse populations or dynamic real-world environments.

## 5. Conclusions

This study systematically evaluated the predictive capabilities of two physiological modalities (i.e., EDA and ECG) using multiple machine learning regression models to assess cybersickness severity in VR environments. Notably, EDA measures achieved superior model performance, reflecting their strong association with sympathetic nervous system activity and their practical value for real-time monitoring. Although integrating ECG with EDA in certain nonlinear models yielded modest performance improvements, multimodal fusion did not always enhance prediction accuracy, emphasizing the need for careful modality selection and fusion strategies. Importantly, SHAP-based interpretability analysis revealed distinct roles of key features such as SC mean, SC var, and SDNN, providing insights into autonomic regulation and the mechanisms underlying cybersickness. These findings advance our understanding of the physiological mechanisms underlying cybersickness and offer a strong empirical foundation for developing efficient, personalized cybersickness prediction and intervention systems in future VR applications.

## Figures and Tables

**Table 1:** The features extracted in the study and used for modeling.

	Extracted Features	Unit	Descriptions	Features Used for Modeling		
				ECG-Based Modeling	EDA-Based Modeling	ECG + EDA-Based Modeling
ECG						
	SDNN	ms	Standard Deviation of NN Intervals (Overall heart rate variability)	√		√
	pNN20	%	Percentage of NN20 Intervals (Indicator of short-term heart rate variability)	√		√
	CSI	--	Cardiac Sympathetic Index (Indicator of autonomic nervous system balance)	√		√
	HRMAD	ms	Median Absolute Deviation of Heart Rate (Robust indicator of heart rate fluctuation)	√		√
	EllipseArea	ms^2^	Area of Ellipse (Area of Poincaré plot ellipse, reflects overall heart rate variability)	√		√
	pNN50	%	Percentage of NN50 Intervals (Indicator of short-term heart rate variability)	√		√
	SD2	ms	Long-term Heart Rate Variability (Long axis of Poincaré plot ellipse, reflects long-term heart rate variability)	√		√
	HR	bpm	Heart Rate (Beats per minute; indicator of heart activity frequency)	√		√
	SDSD	ms	Standard Deviation of Successive Differences (Indicator of short-term heart rate variability)	√		√
	RMSSD	ms	Root Mean Square of Successive Differences (Indicator of parasympathetic nervous system activity)	√		√
	IBI	ms	Inter-Beat Interval (Indicator of heart rhythm)			√
	BR	bps	Breathing Rate (Breaths per second; indicator of respiratory frequency)			
	SD1	ms	Short-term Heart Rate Variability (Short axis of Poincaré plot ellipse, reflects instantaneous heart rate variability)			
EDA						
	SC mean	µS	Skin Conductance Mean (Reflects overall arousal level)		√	√
	SC var	(µS)^2^	Skin Conductance Variance (Indicates spontaneous fluctuation intensity)		√	√
	SC range	µS	Skin Conductance Range (Reflects peak-to-peak amplitude)		√	√
	SCL	µS	Skin Conductance Level (Indicates baseline tonic arousal)		√	√
	SC min	µS	Skin Conductance Minimum (Minimum recorded SCL value)		√	√
	SC max	µS	Skin Conductance Maximum (Maximum recorded SCL value)		√	√
	SC std	µS	Skin Conductance Standard Deviation (Reflects fluctuation magnitude)			√

**Table 2:** Evaluation metrics of different modalities and algorithms.

Modalities	Algorithm	MAE	MSE	RMSE	R^2^
ECG	Linear Regression (LR)	0.71	0.78	0.88	0.33
	Decision Tree (DT)	0.61	0.69	0.83	0.41
	Ensemble Learning (EL)	0.57	0.57	0.75	0.51
	Gaussian Process Regression (GPR)	0.55	0.55	0.74	0.53
	Neural Network (NN)	0.60	0.62	0.79	0.47
	Kernel-based Regression (KR)	0.64	0.69	0.83	0.41
EDA	Linear Regression (LR)	0.82	0.9	0.95	0.22
	Decision Tree (DT)	0.03	0.04	0.19	0.97
	Ensemble Learning (EL)	0.04	0.02	0.15	0.98
	Gaussian Process Regression (GPR)	0.09	0.05	0.22	0.96
	Neural Network (NN)	0.36	0.34	0.59	0.71
	Kernel-based Regression (KR)	0.62	0.6	0.78	0.48
ECG + EDA	Linear Regression (LR)	0.61	0.59	0.77	0.5
	Decision Tree (DT)	0.17	0.22	0.47	0.81
	Ensemble Learning (EL)	0.2	0.15	0.39	0.87
	Gaussian Process Regression (GPR)	0.29	0.2	0.45	0.82
	Neural Network (NN)	0.38	0.29	0.54	0.75
	Kernel-based Regression (KR)	0.48	0.47	0.68	0.60

## Data Availability

The data presented in this study are available on request from the corresponding author. The data are not publicly available due to privacy issues.
